# Can we still stop the migration of physicians from Austria? An evaluation of clinical internships by students of the Medical University of Vienna

**DOI:** 10.1007/s00508-016-1095-5

**Published:** 2016-10-19

**Authors:** Tamara Seitz, Bela R. Turk, Henriette Löffler-Stastka

**Affiliations:** 1 Medizinische Universität Wien, Vienna, Austria; 2 Universitätsklinik für Psychoanalyse und Psychotherapie, Medizinische Universität Wien, Währinger Gürtel 18–20, 1090 Vienna, Austria

**Keywords:** Medical internship, Lack of physicians, Medical students, Medical University of Vienna, Anamnesis

## Abstract

The increasing emigration of graduates of the Medical University of Vienna presents a serious problem. This study examined students’ evaluation of clinical rotations, their self-rated performance, and where they felt the most deficits exist. Medical students answered an online questionnaire surveying the following aspects: an evaluation of their internship; supervision; integration in the team and improvement of field-specific knowledge; the qualities of taking a patient’s medical history by empathy; patient-centeredness; structure; target orientation; and the ability to integrate field-specific knowledge into anamnesis. The data collected indicate that rotations in Austria, especially in Vienna, were evaluated significantly worse than those abroad. Particularly the lack of supervision and integration in the team were criticized. These data stress a dire need for the reform of curricular structures during clinical rotation in the latter years of medical education.

## Introduction

Medical students, as future health-care practitioners, undergo rigorous training in hospital clinics in order to prepare for the daily routine as a physician. The Medical University of Vienna supports a three-tiered approach in integrating medical students into the clinical work routine:

1. Clerkships (*Famulatur*) throughout the entire program amounting to 12 weeks
2. Rotations (*Tertiale*) during the fourth and fifth year, 5 weeks per department for half a day
3. Internship (clinical practical year; *Klinisch Praktisches Jahr*), during the entire sixth year for 16 weeks of each surgical, internal, and free-choice departments

Additionally, medical students may be eligible for a long-term training (*Dauerfamulatur*) throughout the length of their study, independently integrating themselves into departments, commonly with a focus on both clinical practice and research. Students can choose how often and when they join under the condition that they stay there for at least 10 h per month.

Each clinic has a registered training physician responsible for supporting students throughout their clinical training.

The goal of clinical training is not only to learn clinical skills and integrate medical knowledge in practice, but also to develop communication paradigms that support taking a precise, patient-oriented, and hypothesis-driven medical history. Practicing these skills is especially important as up to 80 % of diagnoses are made during initial patient contact [1] and patient satisfaction is highly correlated with physician communication skills [2–6].

Medical students are also integrated into the clinical care team, allowing for the evaluation of whether or not the discipline, the department, or even the country may be suited to them in the future. This aspect has become especially critical as a large and increasing number of graduate medical doctors emigrate from Austria [7], a problematic situation since over 40 % of primary care physicians are at least 55 years of age [8, 9] and hospitals are projected to require 20 % more physicians by 2030 [9].

This survey assessed students’ ratings of their clinical clerkships, investigating differences between Austrian counties in order to identify resources and deficits. Improving the current clinical training regimen may ameliorate the emigration of Austrian medical graduates.

## Methods

For the survey, a student-rated questionnaire was developed assessing completed clinical training. The first half of the questionnaire collects demographic data, type of clinical training (clerkship, rotation, or internship), location, department, length of training, and frequency of taking medical histories. The second half requires students to rate on a scale of Grade A (1 – very good), Grade B (2 – good), Grade C (3 – satisfactory), Grade D (4 – sufficient), and Grade E (5 – insufficient) the following aspects of their training: General assessment of clinical training, supervision by medical professionals and physicians, integration into the clinical team, interest in the medical discipline, improvement of clinical knowledge, improvement of communication skills in taking a medical history, empathy and patient orientation, precision and structure, and the ability to integrate clinical knowledge into the anamnesis.

### Participants

Medical students in the third, fourth, fifth, and sixth academic year (*n* = 3014, 744–763 per year group) at the Medical University of Vienna received an e‑mail with the link to the online survey/questionnaire and instructions. Participation was anonymized; study protocols were approved by the Data Protection Committee and the Ethics Committee of the Medical University of Vienna. Preclinical medical students in the first 2 years of study were excluded.

### Aims

The aim of the study was to determine students’ ratings of clinical training. Correlations between type, length, or location of clinical training and student rating of subcategories as described in the methods section may allow for the development of current curricular frameworks in improving student satisfaction and performance as well as ameliorating emigration of medical graduates.

### Statistical analysis

Spearman’s correlation was used in determining the strength between length of training in weeks, age, and student-rated subcategories. As commonly utilized in social sciences, a correlation coefficient of *r* = 0–0.2 may show a very weak correlation, *r* = 0.2–0.4 a medium correlation, *r* = 0.4–0.7 a strong correlation, and *r* = >0.7 a very strong correlation. Gender analysis was performed using *t* tests and a 5 % significance level was assumed for all tests. Analyses were performed using IBM SPSS Statistics for Windows Version 20.0.

## Results

A total of *n* = 304 students (response rate of 10.1 %) completed the questionnaire, balanced between gender (male/female 52.3 %/47.7 %) and with a median age of 25 years (20–42 years).

In all, 17.5 % (53) of participants reported on their internship year (clinical practical year), 14.8 % (45) reported on clinical rotations, and 62.8 % (191) reported on clerkships; additionally, 4.9 % (15) reported on long-term training. Of the training, 52 % took place in Vienna, 32.6 % in other Austrian counties, and 13.2 % in European cities. Only five students reported on training outside Europe (North America, South America, and Asia).

On average students took medical histories three to four times per week: 51.5 % (154) of participants took one to three histories, 11.7 % (35) four to six histories, and 14.7 % (44) more than six histories per week; 22.1 % (66) took no medical histories during their clinical training.

The students’ general assessment of their clinical training was graded at 21.2 % (64) Grade A [1], 28 % (85) Grade B [2], 16.5 % (50) Grade C [3], 15.8 % (48) Grade D [4] and 18.5 % (56) Grade E [5]. The average grade for clinical training was 2.8 (Grade C+). Subcategory ratings are shown in Table 1.

**Table 1:** Assessment of subcategories, from highest rated to lowest (A–E, 1–5)

Subcategory	Rating	Average rating
Improvement of clinical knowledge	B−	2.24
Improvement of structure and precision in taking a medical history	C+	2.72
Improvement of the ability to integrate clinical knowledge in taking a medical history	C+	2.75
Improvement in empathy and patient orientation in taking a medical history	C+	2.89
Integration into the clinical team	C+	2.93
Supervision by medical professionals and physicians	C+	2.98

Only 36.3 % of participants had a dedicated supervisor. The general assessment of each type of training (clerkship, rotation, internship) is shown in Table 2. The highest-rated subcategory for all participants was “Improvement of clinical knowledge” (rated between Grade B+ and C+; 1.63–2.9), the worst two rated subcategories were “Integration into the clinical team” and “Supervision by medical professionals” (Grade B− to D+; 2.13–3.8).

**Table 2:** General assessment of clinical training by type, from highest rated to lowest (A–E, 1–5)

Type of clinical training	Grade	Average rating
Long-term training	B−	2.2
Internship (clinical practical year)	B−	2.4
Clerkship	C+	2.8
Rotation	D+	3.7

Owing to a small sample size of participants doing their clinical training overseas, results of participants training in Asia and North/South America are assumed to have low validity. Excluding these, the best-rated subcategories for all locations (Table 3) were “Improvement of clinical knowledge,” and the worst in Austria (including Vienna) were “Integration into the clinical team” and “Supervision by medical professionals.” Training in other parts of Europe saw “Improvement of empathic and patient-centered history taking skills” as the lowest-rated subcategory.

**Table 3:** General assessment of clinical training by location, from highest rated to lowest (A–E, 1–5)

Location of training	Grade	Average rating
South America	B	2
Europe (excluding Austria)	B−	2.3
Austria (excluding Vienna)	C+	2.6
North America	C+	2.7
Asia	C	3
Vienna	C−	3.1

Students’ general assessment of clinical training strongly correlated with the duration of training in weeks (*r* = −0.94, *p* = 0.001), significantly improving the longer the training lasted. Correlations between length of training and subcategories are shown in Table 4.

**Table 4:** Correlations between duration of clinical training and rating of subcategories, from highest correlated to lowest

Subcategory	Correlation coefficient *r*	*p*	Strength of correlation
Improvement of structure and precision in taking a medical history	−0.3	0.0001	Medium
Improvement of the ability to integrate clinical knowledge in taking a medical history	−0.27	0.0001	Medium
Improvement in empathy and patient orientation in taking a medical history	−0.26	0.0001	Medium
Integration into the clinical team	−0.21	0.0001	Medium
Supervision by medical professionals and physicians	−0.18	0.02	Low
Improvement of clinical knowledge	−0.17	0.004	Low

Neither age nor gender showed statistical influence on students’ rating both in general assessment and for subcategories. The influence of students’ interest in the specific field on average ratings and rating categories is shown in Table 5.

**Table 5:** Correlation between general assessment of clinical training and subcategories including students interest in the specific medical field, from highest correlated to lowest

Subcategory	Correlation coefficient *r*	*p*	Strength of correlation
Integration into the clinical team	0.71	0.0001	Very strong
Supervision by medical professionals and physicians	0.7	0.0001	Very strong
Improvement of clinical knowledge	0.6	0.0001	Strong
Improvement of the ability to integrate clinical knowledge in taking a medical history	0.59	0.0001	Strong
Improvement of structure and precision in taking a medical history	0.58	0.0001	Strong
Improvement in empathy and patient orientation in taking a medical history	0.55	0.0001	Strong
Interest in the specific medical field	0.33	0.0001	Medium

## Discussion

The low ratings of clinical training by medical students at the Medical University of Vienna stress the necessity of revising clinical training frameworks, especially clinical training in Viennese hospitals. We propose the following systematic frameworks in order to ameliorate low-rated subcategories:

1. *Establishing a Competency Catalog for clinical training:* Each student must successfully complete a defined number of case-based competencies by the end of their training, specific for each medical field and department. The competencies should be trained in patient contact, using evidence-based clinical methods. An Austrian-wide Competency Catalog would standardize training practices and ameliorate skill deficits. A physician supervisor would take responsibility for students’ completion of the Competency Catalog.
2. *Introducing an openly accessible assessment system for training hospitals:* A visible rating system would allow students and residents to identify teaching hospitals more suited toward their needs and motivate other departments to raise their training standards to combat a shortage of doctors.
3. *Improving the role of dedicated supervisors and physician educators: *Supervisors’ workload and performance may be improved via professional incentives including: financial incentive, compensatory continued medical education credits (*Fortbildungspunkte*), or allotted time off as compensation. Improving the role of educators may be facilitated by replacing supervisors who receive multiple negative reviews (see point 2) or fail to supervise the completion of a student’s Competency Catalog (See point 1).
4. Longer durations for clinical training.
5. *Revising clinical rotations:* The authors support the transfer of declarative to associative procedural clinical knowledge (from textbook to bedside), especially during clinical training. Lectures allow students to generate large amounts of declarative knowledge, tested in yearly SIP exams, while smaller group seminars allow for individual feedback in understanding complex concepts [10, 11]. Translating explicit knowledge into clinical procedure, however, requires students to first be integrated into a clinical care group and begin to assume a role (for example, in taking medical history) where procedural knowledge (a structured anamnesis) is integrated and associated with declarative knowledge (symptoms of specific diseases leading to ask different questions).

As preparation for clinical training, mandatory completion of online cases utilizing “case-based blended learning” is being implemented [12, 13]. This program has seen high student ratings [14] since its recent implementation in 2013/2014. Interactive cases support the transfer of declarative knowledge (requiring students to correctly recognize symptoms in a case report), to procedural knowledge (asking the correct questions to elicit a working hypothesis, defining next diagnostic and therapeutic steps).

We recommend testing medical students’ procedural skills after each clinical rotation; this will have the benefit of verifying and improving clinical skills and clinical decision-making, motivating the student and identifying deficits. In addition, more frequent examinations may improve scores and decrease stress during the preparation for end-of-year exams via cumulative learning [15].

6. *Establishing a Student E-portfolio:* We recommend a portfolio that lists which skills and competencies a student has gained throughout their entire course of study. These may be tested following completion during regular examinations (e. g., Objective Structured Clinical Examinations, progress reports, etc.). This may allow students to identify their individual strengths and weaknesses. In addressing specific deficits, opportunities to re-visit previous content or classes may facilitate the further development of learning paradigms. Additionally, active participation by the student in their education process may improve student performance and aid in deciding on which residency or postgraduate training to take.

## Conclusion

In summary, clinical training of medical students from the Medical University of Vienna during clerkships, rotations, and in their internship year in Austria, especially in Vienna, is rated worse than in other European countries. Clinical rotations (in the fourth to fifth year) may require a comprehensive revision.

Comparisons of curricula between other medical universities may allow for the identification of strategies for implementing changes to the clinical training framework. Additionally, a cost–benefit analysis should be performed to implement these suggested changes. The authors endeavor to improve the medical education of students from the Medical University of Vienna in order to combat the emigration of medical graduates.

A potential limitation of this study is that the participants might not be representative of all of the medical students at the Medical University of Vienna, although we cooperated closely with the students’ council and students’ representatives. Nevertheless, further investigations would be appreciated.
